# Virus Caused Imbalance of Type I IFN Responses and Inflammation in COVID-19

**DOI:** 10.3389/fimmu.2021.633769

**Published:** 2021-04-12

**Authors:** Jintao Zhang, Chunyuan Zhao, Wei Zhao

**Affiliations:** ^1^ Department of Immunology, School of Basic Medical Science, Cheeloo College of Medicine, Shandong University, Jinan, China; ^2^ State Key Laboratory of Microbial Technology, Shandong University, Jinan, China; ^3^ Department of Cell Biology, School of Basic Medical Science, Cheeloo College of Medicine, Shandong University, Jinan, China

**Keywords:** COVID-19, SARS-CoV-2, type I interferons, inflammation, innate immunity

## Abstract

The global expansion of coronavirus disease 2019 (COVID-19) caused by severe acute respiratory syndrome coronavirus 2 (SARS-CoV-2) has emerged as one of the greatest public health challenges and imposes a great threat to human health. Innate immunity plays vital roles in eliminating viruses through initiating type I interferons (IFNs)-dependent antiviral responses and inducing inflammation. Therefore, optimal activation of innate immunity and balanced type I IFN responses and inflammation are beneficial for efficient elimination of invading viruses. However, SARS-CoV-2 manipulates the host’s innate immune system by multiple mechanisms, leading to aberrant type I IFN responses and excessive inflammation. In this review, we will emphasize the recent advances in the understanding of the crosstalk between host innate immunity and SARS-CoV-2 to explain the imbalance between inflammation and type I IFN responses caused by viral infection, and explore potential therapeutic targets for COVID-19.

## Introduction

The global expansion of coronavirus disease 2019 (COVID-19) caused by severe acute respiratory syndrome coronavirus 2 (SARS-CoV-2) has emerged as one of the greatest public health challenges and imposes a considerable threat to human health. Similar to SARS-CoV and MERS-CoV infection, SARS-CoV-2 infection frequently induces high levels of proinflammatory cytokines, leukocyte changes, high D-dimer, and increased lactate dehydrogenase levels (1–3). COVID-19 exhibits varied clinical manifestations, ranging from fever, cough, diarrhea, and fatigue to pulmonary edema, septic shock, multiple organ failure, and even death. The multiple symptoms indicate that COVID-19 is a systemic inflammatory disorder rather than a single respiratory disease (2).

Innate immunity functions as the first line of defense for the host to eliminate invading viruses through the initiation of type I IFNs (including IFN-α and IFN-β)-dependent antiviral responses and induction of inflammation (4). As a bridge, it initiates antiviral adaptive immune responses and controls the intensity of adaptive immunity. Therefore, optimal activation of innate immunity and balanced type I IFN responses and inflammation are beneficial for efficient elimination of invading viruses. However, excessive inflammation caused by viral invasion can induce excessive production of inflammatory cytokines and initiate acute respiratory distress syndrome (ARDS), which is associated with the increased risk of death (1, 5–7). In contrast to heightened levels of inflammation, reduced or delayed type I IFN responses accompanied by relatively high viral titers have been observed in COVID-19 patients, particularly in critical patients in intensive care units (ICU) (8, 9). While each cell population of patients with mild COVID-19 displays a coordinated interferon signature, those with severe cases are thought to dampen IFN responses through CD32b (10). Recent evidence from two translational medical researches similarly highlighted the importance of type I IFN. They confirmed that defective IFN signaling stands as the main causes of serious COVID-19 besides advanced age and underlying diseases and may account for nearly 13% of severe cases (11, 12). Imbalance between inflammation and IFN I responses indicates poorer prognosis for COVID-19 patients, and worse disease outcomes. In this review, we will emphasize recent advances in understanding the crosstalk between host innate immunity and SARS-CoV-2, and explain the imbalance between inflammation and IFN I responses to explore potential therapeutic targets for COVID-19.

## Entry of SARS-CoV-2

**Figure 1:**
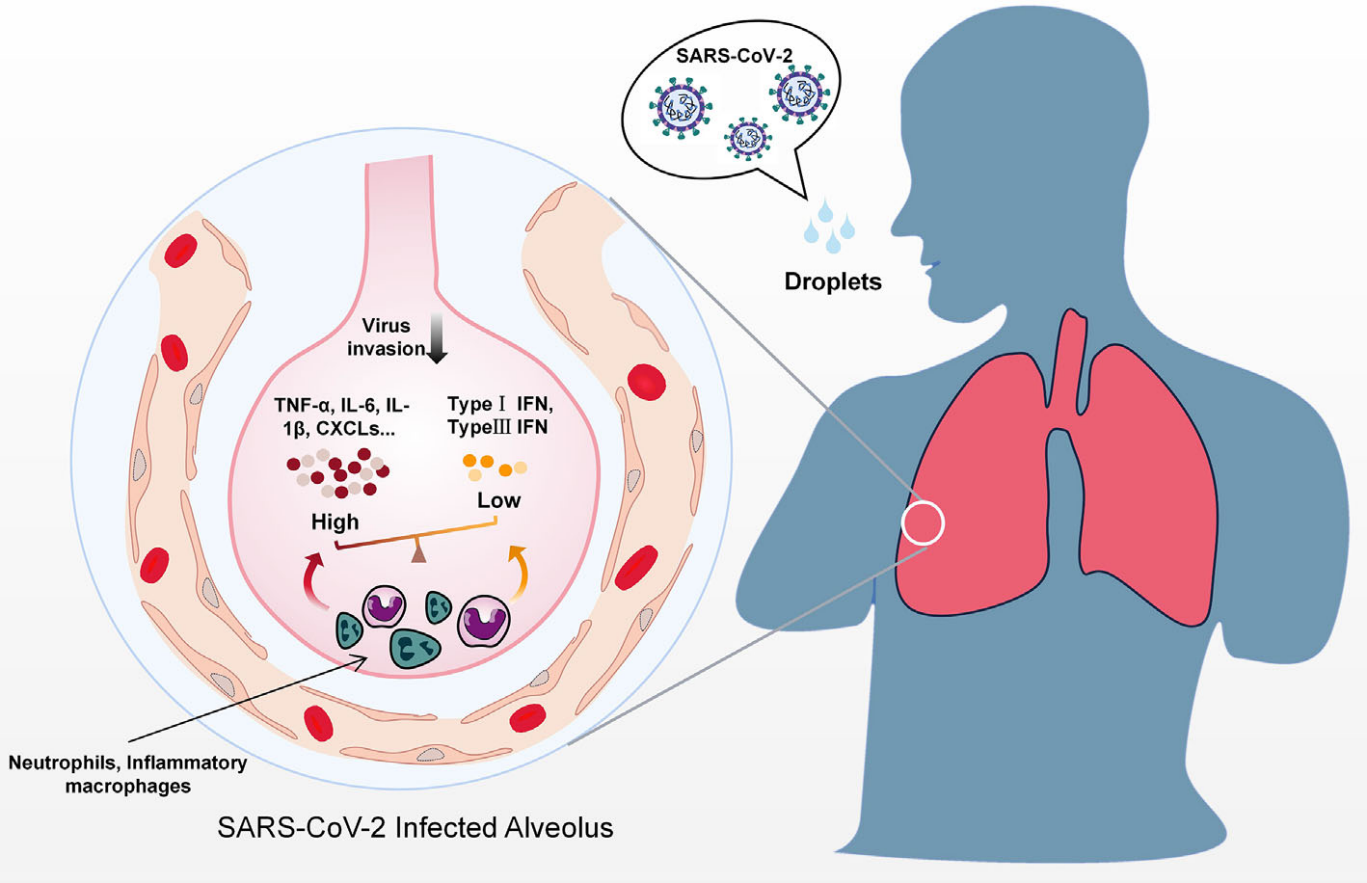
Predicted immune dysregulation in the lung during SARS-CoV-2 infection. After contact with droplets containing SARS-CoV-2, host immune responses are activated in the lung. Activation of immune cell subgroups such as inflammatory macrophages and neutrophils results in the secretion of massive amounts of inflammatory cytokines, including TNF-α, IL-6, IL-8, and CXCLs. In contrast to excessive proinflammatory cytokines, IFNs levels are lower during the early phase of infection.

Like many other respiratory coronaviruses, SARS-CoV-2 mainly infects the host *via* the respiratory tract and is transmitted *via* respiratory droplets (13) (**Figure 1**). The SARS-CoV-2 genome is approximately 29.7 kb long and encodes four structural proteins (spike protein S, envelope protein E, membrane protein M, and nucleocapsid protein N), 16 non-structural proteins (NSPs), and 9 accessory proteins (14–16), which share high sequence similarity to their SARS-CoV counterparts.

Among all the viral proteins, the S protein initiates the infection process by mediating the attachment of the virus to host cells through angiotensin-converting enzyme 2 (ACE2) (13). ACE2 mainly exists in the intestine, heart, kidneys, and testes, and it is correlated with the distribution of SARS-CoV-2 (17). According to single-cell RNA sequencing (scRNA-Seq), the expression of ACE2 in the lungs was primarily observed in the alveolar type II cells (AT2), which are probably the primary target of SARS-CoV-2 (18). The latest longitudinal study in conjunction with airway and blood samples also indicates that severe inflammation during SARS-CoV-2 infection is mainly driven by cytokines in the lung rather than systemic (19). Even worse, the SARS-CoV-2-induced IFN I responses also evidently increase the cellular ACE2 levels, which may increase susceptibility to infection (20). In addition, SARS-CoV-2-encoded N protein, NSP7b, and NSP8 all participate in viral replication, evolution, and immune evasion during infection (21, 22). Emerging evidence confirms that both structural and nonstructural proteins of SARS-CoV-2 interfere with host innate immune responses and participate in the pathogenesis of COVID-19 (14, 23).

## Innate Immune Responses Against SARS-CoV-2

**Figure 2:**
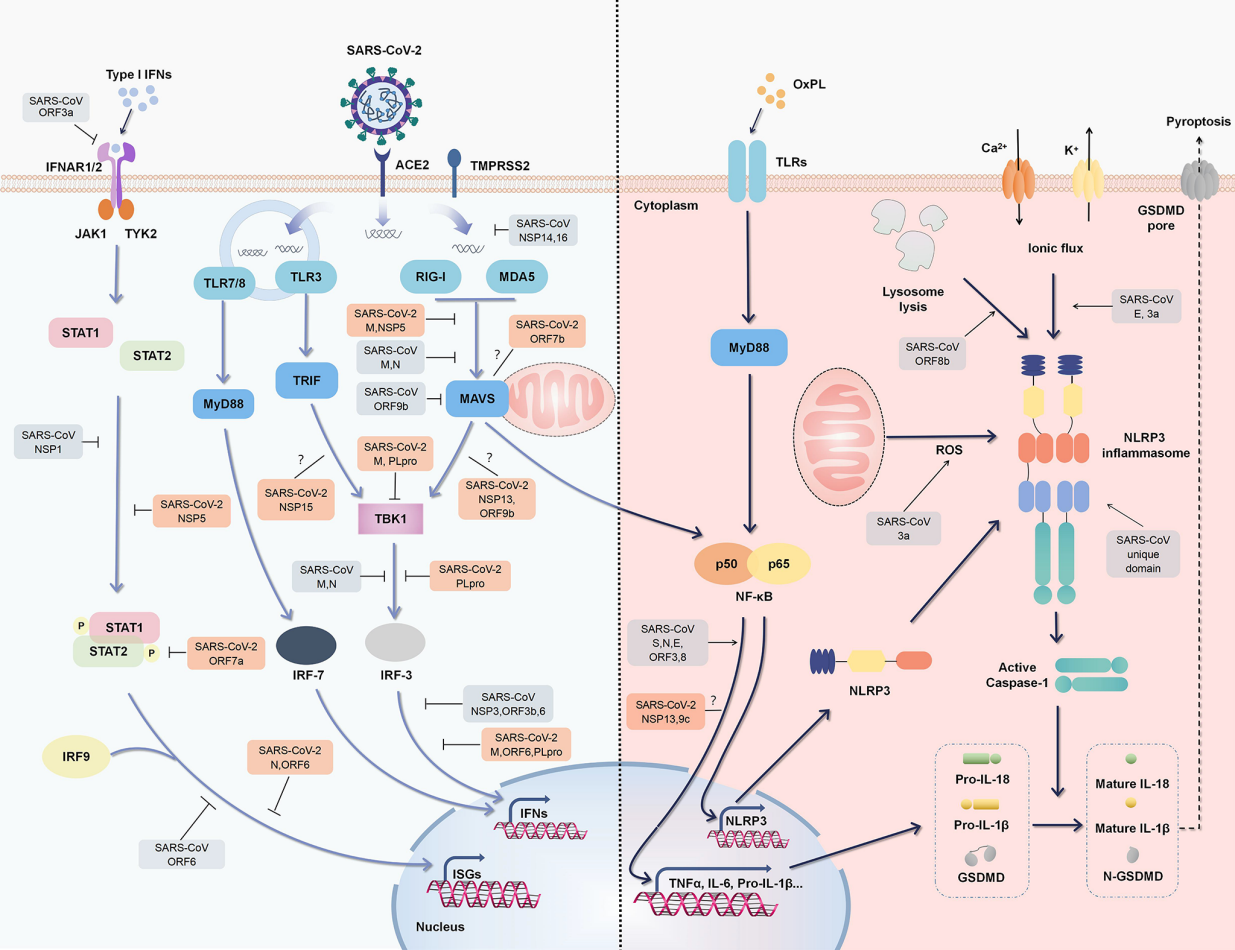
Regulation of type I IFN responses and inflammation by SARS-CoV-2 and SARS-CoV. An outline of IFN signaling (left) and major inflammatory signaling (right) is shown, annotated with the known mechanisms by which SARS-CoV activates or suppresses signals (gray). Some SARS-CoV-2 proteins have also been confirmed or speculated to interact with these pathways (orange).

To effectively defend against viruses, host cells initiate antiviral innate immune responses by producing a number of IFNs and other proinflammatory cytokines. IFNs and downstream interferon-stimulated genes (ISGs) play fundamental roles in limiting viral replication (24) (**Figure 2**). Following invasion, SARS-CoV-2 releases viral RNA and proteins into cells, which are recognized by the host immune system as pathogen associated molecular patterns (PAMPs), thus initiating the secretion of IFNs and antiviral innate immune responses (25). Various pattern recognition receptors (PRRs), including Toll-like receptor 3 (TLR3), TLR7, TLR8, retinoic acid-inducible gene I (RIG-I), and melanoma differentiation-associated protein 5 (MDA5) are candidates for the recognition of SARS-CoV-2, since they are considered to sense viral components; particularly viral RNA (26). Among these PRRs, RIG-I/MDA5 are proven to be the main sensors in MERS-CoV recognition and inflammatory cascade initiation (27). TLR7/8 is also reportedly involved in sensing coronaviruses including SARS-CoV (28).

PRR signaling activated by viral components induces nuclear factor (NF)-κB activation, which triggers the expression of a large number of proinflammatory cytokines. Some of the metabolites as oxidized phospholipid (OxPL) are also increased during coronavirus infection, further triggering NF-kB activation *via* TLR4 (29). NF-κB activation induces the expression of nucleotide-binding oligomerization domain-, leucine-rich repeat- and pyrin domain-containing 3 (NLRP3), pro-IL-1β, and pro-IL-18 (the priming stage of the NLRP3 inflammasome). Subsequently, cellular damage or distress caused by viral infection, leading to the accumulation of reactive oxygen species and ion fluxes (damage associated molecular patterns, DAMPs), results in the activation stage of NLRP3 inflammasome (30). NLRP3 inflammasome activation then mediates the maturation of IL-1β, IL-18, and the propyroptotic factor gasdermin D (GSDMD), and thus induces inflammation-associated cell death known as “pyroptosis” and further amplifies host inflammatory responses (30, 31) (**Figure 2**).

The activation of multiple PRRs facilitates the establishment of antiviral states by recruiting multiple adaptor proteins (e.g., mitochondrial antiviral signaling protein (MAVS), TANK binding kinase 1 (TBK1), tumor necrosis factor receptor-associated factor (TRAF) 3 and TRAF6) and inducing a variety of cytokines. On this basis, different immune cells are recruited to infection sites and initiate adaptive immune responses (32). Although substantial IFN I responses and optimal inflammation are beneficial for the eradication of invading viruses, an imbalance between type I IFN responses and inflammation induced by different viral proteins can cause numerous detrimental effects (**Figure 1**). In a SARS-CoV-2 infection hamster model, significant inflammation occurred in distal tissues even while productive SARS-CoV-2 replication was low (33). In the late phase of infection, a large increase in delayed type I IFN responses occurs, which induces the secretion of proinflammatory cytokines such as C-X-C motif chemokine ligand 10 (CXCL10) found in pulmonary autopsy tissues, and recruits and activates inflammatory monocyte-macrophages, resulting in the impairment of T cell responses and lung immunopathology (34, 35). A similar situation was also revealed when single-cell RNA-seq was performed using peripheral blood mononuclear cells obtained from severely affected COVID-19 patients (36). In addition to type I IFNs, type II IFN (IFN-γ) which is mainly secreted by T cells and natural killer cells was also increased in the serum of COVID-19 patients (37). In early SARS-CoV-2 infection increased IFN-γ may contribute to antiviral immunity in multiple ways, including the promotion of antigen presentation, inflammatory cell activation and even by directly stimulating the expression of multiple ISGs (38). As the disease progresses however, the viral infection may destroy T-cells and reduce the production of IFN-γ.

## Delayed IFN Responses in SARS-CoV-2 Infection

Like SARS-CoV, SARS-CoV-2 is also highly sensitive to IFNs *in vitro* (35, 39). In a hamster infection model, intranasal IFN I effectively inhibited SARS-CoV-2 replication and transmission (33). In a phase 2 COVID-19 trial, a combination including IFN-β1b effectively alleviated symptoms and shortened the duration of viral shedding, indicating the critical role of IFNs during SARS-CoV-2 infection (40). Aging and underlying diseases have a potentially negative impact upon IFN production (41–43). Consistent with this, in multiple retrospective cohort studies there have been higher fatalities in elderly patients with severe COVID-19 (2, 3). Similarly, in macaques pulmonary injury was more prominent in an aged group than in a group of young adults (41).

Genetic mutations (TLR3-, TLR7- and IRF7-dependent) and neutralizing auto-antibodies (Abs) that influence type I interferon signaling were shown in severe COVID-19 patients (11, 12, 44). All these findings underscore the importance of IFNs for protection against SARS-CoV-2. During early SARS-CoV-2 infection however, the host type I IFN response can evidently be low and insufficient, which may be partly caused by immune escape mechanisms of viruses. In a longitudinal follow-up study including 32 COVID−19 patients and 16 influenza-associated pneumonia patients who had similar clinical characteristics to the COVID-19 patients, SARS-CoV-2, but not influenza virus, could trigger an untuned immune response that presented as suppressed and delayed IFN responses and a persistent inflammatory response (45). Delayed type I IFN signaling not only provides a key window for viral replication but could induce tissue damage as observed in SARS-CoV infection (35, 46).

### Viral Proteins of SARS-CoV-2 Inhibit Type I IFN Secretion

Four non-structural proteins of SARS-COV-2, including NSP13, NSP15, open reading frame (ORF) 7b, and ORF9b, were identified as interactors of host proteins involved in IFN signaling by affinity-purification mass spectrometry (23, 47). At least eight proteins (NSP1, NSP3, NSP12, NSP13, NSP14, ORF3, ORF6, and M) have also been proven to inhibit IFN-β production by gene reporter assays (16). Genetic and clinical data revealed that deletion mutations in NSP1 of SARS-CoV-2 coding region is a variant hotspot that could lead to lower IFN response (48). These studies indicate the existence of a complex regulatory network between SARS-CoV-2 and the host immune system. Viruses tend to downregulate the host immune system by direct disruption of antiviral-associated proteins.

RIG-I activation is dependent on ubiquitination by tripartite motif containing (TRIM)25, of which the interaction could be blocked by NSP5 of SARS-CoV-2 and N protein of SARS-CoV (**Figure 2**) (26, 49). The M protein of SARS-CoV-2 and SARS-CoV prevents the formation of the TRAF3-TBK1/IKK∈ complex, thus suppressing IFNs production (50, 51). SARS-CoV-2 M protein even affects the formation of the RIG-I–MAVS–TRAF3–TBK1 multi-protein complex and subsequent phosphorylation of IFN regulatory factor 3 (IRF3) (51). As well as steric hindrance, viral components also directly target critical adaptors in antiviral immunity. SARS-CoV-2 ORF6 inhibits IFN signals by preventing IRF3 nuclear translocation (16). As a key protease of SARS-CoV-2 that regulates viral replication and spread, papain-like protease (PLpro, a part of NSP3) attenuates IFN responses *via* the cleavage of ISG15 from IRF3 (52). SARS-CoV-2 also affects the host through the translational level. NSP1 binds to 40S ribosomal subunits and obstructs host mRNA translation, thus effectively inhibiting RIG-I-mediated IFN responses (**Figure 2**) (53).

Although a large number of regulatory proteins have been identified *via* high-throughput screening, their underlying mechanisms with respect to relationships with SARS-CoV-2 are poorly understood. Fortunately, there are great similarities in sequence between SARS-CoV-2 and SARS-CoV, and continued comparison may reveal additional useful information regarding regulation. The N7-methylguanosine (m7G) cap is the defining structural feature of eukaryotic mRNA that distinguishes it from viral RNA. SARS-CoV NSP14 is a novel cap N7-methyltransferase that processes the cap structure of viral RNA to mimic host mRNA and evade recognition (54). ORF9b degrades MAVS, as well as TRAF3 and TRAF6, by usurping poly(C)-binding protein 2 and AID4, a gatekeeper and E3 ligase, thus controlling MAVS expression levels (55). As a crucial adaptor of the antiviral signaling pathway, IRF3 is also a key target of multiple SARS proteins including PLpro, N, ORF3b, and ORF6 protein (**Figure 2**) (56, 57).

### SARS-CoV-2 Proteins Inhibit the JAK-STAT Pathway

As a downstream signaling pathway of IFN, JAK-STAT pathway is crucial for type I IFNs triggered ISGs expression and antiviral responses. During viral infection, responder cells produce and secrete type I IFNs which subsequently bind to their receptor IFNAR (IFN-α/β receptor) to initiate JAK-STAT signaling cascades (58). Both SARS-CoV-2 and SARS-CoV infection affect JAK-STAT signaling, thus inhibiting host antiviral innate immunity independent of the level of IFNs (16, 49, 59–64). Up to now, at least four SARS-CoV-2 proteins (NSP5, ORF7a, N, ORF6) and three SARS-CoV proteins (ORF3a, NSP1, ORF6) have been proved to directly affect the activation of JAK-STAT pathway by multiple mechanisms including mediating STAT1/2 degradation and suppressing their phosphorylation and nuclear translocation (16, 49, 59–64). Resultantly, the expression of ISGs reduced, and antiviral effects get impaired.

## Excessive Inflammation Caused by SARS-CoV-2

During SARS-CoV-2 infection, aberrant inflammatory cytokine responses and the induction of a cytokine storm is closely associated with extensive lung damage and disease severity (65). Compared to mild-to-moderate affected patients, in these severe cases higher levels of pro-inflammatory cytokines (including IL-6, IL-10, and TNF-α, etc.) are secreted, and their secretion is correlated with a high serum titer of SARS-CoV-2 and an increased risk of death (66–68). The disrupted secretion of proinflammatory and anti-inflammatory cytokines in severely ill patients results in vascular leakage and fluid accumulation, which is the main cause of ARDS (69). Thus, the seriousness of clinical symptoms may be highly correlated with the inflammatory status of COVID-19 patients.

### NF-κB-Associated Inflammation

Abnormal NF-κB activation is vital for the initiation and progression of multiple inflammatory respiratory diseases and ARDS (70, 71). For SARS-CoV, the S, N, and E proteins as well as ORF3 and ORF8 can activate NF-κB signaling, leading to the secretion of proinflammatory cytokines (72–75). During SARS-CoV-2 infection, two non-structural proteins, NSP13 and ORF9c interact with NF-κB signaling proteins (TLE1, TLE3, TLE5, NLRX1, F2RL1, and NDFIP2), suggesting that SARS-CoV-2 can regulate the NF-κB signaling pathway (23). The vasoconstrictor angiotensin II (AngII) is also a key factor involved in the pro-inflammatory responses. As the substrate of ACE2, AngII overexpresses after S protein-induced ACE2 internalization (76). After binding with angiotensin receptor 1 (AT1R), AngII initiates numerous kinase activations that result in subsequent inflammatory factor production (76).

### NLRP3 Inflammasome-Associated Inflammation

Higher numbers of NLRP3 and ASC puncta have been observed in COVID-19 patients, and IL-18 and Caspase-1 p20 levels are correlated with disease severity and clinical outcome (77). Specifically, ion fluxes, protein aggregates, and reactive oxygen species (ROS) are all activators of the NLRP3 inflammasome, which can be induced by viral replication and proliferation (30). For SARS-CoV, the E protein activates the NLRP3 inflammasome by forming an ion channel in host ERGIC/Golgi membranes, and inducing Ca^2+^ ionic fluxes (78). Similarly, SARS-CoV 3a induces K^+^ efflux and mitochondrial ROS *via* ion channel activity (31). ORF8b activates the NLRP3 inflammasome by forming an insoluble complex, as well as direct binding with NLRP3 (79). The E protein, with its unique domain containing three macrodomains (N, M, and C), promotes the production of several cytokines, including IL-1β, TNF-α, IL-6, CXCL10, and C-C motif chemokine ligand (CCL)5, in a NLRP3-dependent manner (78, 80). Such positive feedback loop induced by a pro-inflammatory cytokine eventually results in ARDS, which is a primary contributor to the development of SARS-CoV-induced pulmonary inflammation (30, 81). Moreover, activation of the inflammasome can consequently induce cellular pyroptosis and further aggravate the inflammatory process, leading to extensive tissue inflammation and damage (30). Thus, the NLRP3 inflammasome plays a key role in ARDS and cytokine release syndrome. With the discovery of regulatory proteins for SARS-CoV-2, thorough studies will be conducted to determine the mechanisms governing inflammasome activation.

## Potential Therapeutic Targets Based on the Virus-Host Interaction

COVID-19 has rapidly caused a worldwide health crisis, partly because SARS-CoV-2 infects people regardless of age, sex, and race, and no specific antiviral drugs are currently available. Given this situation, effective and specific drugs need to be developed urgently. Innate immune responses in COVID-19 patients can be divided into two phases-early phase (inhibited antiviral innate immunity and low levels of IFNs) and late phase (amplified innate immunity and high levels of IFNs and proinflammatory cytokines), and treatment needs to be administered carefully. Serum IFNs and viral loads need to be detected to determine the suitable timing of treatment administration.

During the early phase, emphasis could be put on inducing IFNs and improving antiviral immunity. In a phase 2 trial for the treatment of SARS-CoV-2 infection, patients receiving SNG001 (nebulized IFN-β1a) exhibited greater improvement and more rapid recovery (82). This phenomenon indicated that local administration of IFN might have different significance from systemic administration. The location, time and duration of IFN exposure may be the key parameters to determine the outcome of viral infection. It also suggested that there may be a substantially greater window (more than 7 days) for beneficial IFN I treatment than initially thought (82). Notably however, in some patients who have neutralizing auto-antibodies against IFNs possibly because of recombination-activating genes (RAG) deficiency, IFNs especially IFN-α administration, is likely to be useless (12). Viral PLpro may cleave proteinaceous post-translational modifications on host proteins as an evasion mechanism against host antiviral immune responses. Given this, GRL-0617 could maintain host IFN responses by targeting SARS-CoV-2 PLpro (52). Similarly, a recent study revealed that famotidine, a histamine receptor-2 blocker, could bind and inhibit the SARS-CoV-2 NSP5 and restore the activation of RIG-I and STAT1 as described above (83). Moreover, in a recent retrospective cohort study, the administration of famotidine in hospitalized COVID-19 patients reduces the risks of severe disease outcomes (84). Currently, the randomized controlled trial of famotidine (NCT04370262) is still underway, whose results are awaited with interest (58).

During the late phase, a primary goal of treatment is to maintain inflammation and antiviral immunity at a moderate level. As mentioned above, the activation of NLRP3 inflammasome during COVID-19 could promote excessive release of inflammatory cytokines. To address this, NLRP3 inflammasome inhibitor (Tranilast) in the treatment of COVID-19 is undergoing clinical trial in China (85). Another potential therapeutic agent is resveratrol which has the ability to suppress NF-κB and inhibit NLRP3 inflammasome activation. Existed studies demonstrated that the administration of resveratrol could inhibit SARS-CoV-2 infection *in vitro* and ameliorate the pulmonary inflammation and lung injury induced by respiratory viruses *in vivo* (86, 87). In order to relieve the excessive inflammatory responses, targeted drugs are an extremely important strategy in COVID-19 clinical therapy. Currently, the IL-1 receptor antagonist anakinra, monoclonal antibodies against IFN-γ and IL-6 are all candidates for relieving excess inflammatory responses, and their curative effects in COVID-19 patients were highly anticipated (88, 89).

## Conclusion

In this review, we focused on recent advances in interactions between host immune system and SARS-CoV and SARS-CoV-2, with special emphasis on the imbalance of type I IFNs and inflammation caused by viral infection. At present, IFN therapy was suggested as only beneficial at the early stages of SARS-CoV-2 infection, but has little effect on hospitalized patients. Several recent studies have also found that IFN signaling could interfere with lung epithelial repair during recovery from viral infection, thus aggravating the lung injuries (90, 91). In COVID-19, the role, dosage and time of IFN in the treatment are worth further examination. Better understanding of the crosstalk between host and SARS-CoV-2 will help to optimize treatment regimens and explore more potential therapeutic targets for COVID-19.

## Author Contributions

JZ drafted the manuscript and figures. CZ and WZ edited the manuscript and figures. All authors contributed to the article and approved the submitted version.

## Funding

This work was supported by grants from the National Natural Science Foundation of China (81622030, 31870866, 81861130369, and 81901609). WZ is a Newton Advanced Fellow awarded by the Academy of Medical Sciences (NAF007\1003).

## Conflict of Interest

The authors declare that the research was conducted in the absence of any commercial or financial relationships that could be construed as a potential conflict of interest.
